# Validity of a minimally invasive autopsy for cause of death determination in stillborn babies and neonates in Mozambique: An observational study

**DOI:** 10.1371/journal.pmed.1002318

**Published:** 2017-06-20

**Authors:** Clara Menendez, Paola Castillo, Miguel J. Martínez, Dercio Jordao, Lucilia Lovane, Mamudo R. Ismail, Carla Carrilho, Cesaltina Lorenzoni, Fabiola Fernandes, Tacilta Nhampossa, Juan Carlos Hurtado, Mireia Navarro, Isaac Casas, Paula Santos Ritchie, Sonia Bandeira, Sibone Mocumbi, Zara Jaze, Flora Mabota, Khátia Munguambe, Maria Maixenchs, Ariadna Sanz, Inacio Mandomando, Alfons Nadal, Anna Goncé, Carmen Muñoz-Almagro, Llorenç Quintó, Jordi Vila, Eusebio Macete, Pedro Alonso, Jaume Ordi, Quique Bassat

**Affiliations:** 1 ISGlobal, Barcelona Centre for International Health Research (CRESIB), Hospital Clinic of Barcelona, Universitat de Barcelona, Barcelona, Spain; 2 Centro de Investigação em Saúde de Manhiça, Maputo, Mozambique; 3 Consorcio de Investigación Biomédica en Red de Epidemiología y Salud Pública (CIBERESP), Madrid, Spain; 4 Department of Pathology, Hospital Clinic of Barcelona, Universitat de Barcelona, Barcelona, Spain; 5 Department of Microbiology, Hospital Clinic of Barcelona, Universitat de Barcelona, Spain; 6 Department of Pathology, Maputo Central Hospital, Maputo, Mozambique; 7 Faculty of Medicine, Eduardo Mondlane University, Maputo, Mozambique; 8 Department of Pediatrics, Maputo Central Hospital, Maputo, Mozambique; 9 Department of Gynecology and Obstetrics, Maputo Central Hospital, Maputo, Mozambique; 10 Department of Maternal-Fetal Medicine, BCNatal, Hospital Clinic of Barcelona, Universitat de Barcelona, Barcelona, Spain; 11 Department of Molecular Microbiology, University Hospital Sant Joan de Déu, Barcelona, Spain; 12 Faculty of Medicine, Universitat Internacional de Catalunya, Barcelona, Spain; 13 ICREA, Catalan Institution for Research and Advanced Studies, Barcelona, Spain; Umeå Centre for Global Health Research, Umeå University, SWEDEN

## Abstract

**Background:**

Over 5 million stillbirths and neonatal deaths occur annually. Limited and imprecise information on the cause of these deaths hampers progress in achieving global health targets. Complete diagnostic autopsies (CDAs)—the gold standard for cause of death determination—are difficult to perform in most high-burden settings. Therefore, validation of simpler and more feasible methods is needed.

**Methods and findings:**

In this observational study, the validity of a minimally invasive autopsy (MIA) method in determining the cause of death was assessed in 18 stillbirths and 41 neonatal deaths by comparing the results of the MIA with those of the CDA. Concordance between the categories of diseases obtained by the 2 methods was assessed by the Kappa statistic, and the sensitivity, specificity, positive, and negative predictive values of the MIA diagnoses were calculated. A cause of death was identified in 16/18 (89%) and 15/18 (83%) stillborn babies in the CDA and the MIA, respectively. Fetal growth restriction accounted for 39%, infectious diseases for 22%, intrapartum hypoxia for 17%, and intrauterine hypoxia for 11% of stillborn babies. Overall, the MIA showed in this group a substantial concordance with the CDA (Kappa = 0.78, 95% CI [0.56–0.99]). A cause of death was identified in all (100%) and 35/41 (85%) neonatal deaths in the CDA and the MIA, respectively. In this group, the majority of deaths were due to infectious diseases (66%). The overall concordance of the MIA with the CDA in neonates was moderate (Kappa = 0.40, 95% CI [0.18–0.63]). A high percentage of accuracy was observed for the MIA in all the diagnostic categories in both stillbirths and neonates (>75%). The main limitation of this study is that some degree of subjective interpretation is inherent to cause-of-death attribution in both the MIA and the CDA; this is especially so in stillbirths and in relation to fetal growth restriction.

**Conclusions:**

The MIA could be a useful tool for cause-of-death determination in stillbirths and neonatal deaths. These findings may help to accelerate progress towards meeting global health targets by obtaining more accurate information on the causes of death in these age groups, which is essential in guiding the design of new interventions and increasing the effectiveness of those already implemented.

## Abstract 2

Clara Menendez and colleagues examine the validity of a minimally invasive autopsy to determine cause of death in stillborn babies and neonates compared with complete diagnostic autopsy in Mozambique.

## Author summary

**Why was this study done?:**

- Over 5 million stillbirths and neonatal deaths occur annually.
- The vast majority of these deaths (99%) take place in low- and middle-income countries, where vital registration systems are inadequate or nonexistent and rarely medically certified.
- Verbal autopsies, the main source of information in these areas, are subject to a high degree of misclassification errors for conditions with poor clinical specificity such as most peri-neonatal deaths.
- Limited and imprecise information on the cause of these deaths hampers progress in achieving global health targets.
- Complete diagnostic autopsies—the gold standard for cause-of-death determination—are difficult to perform in most high-burden settings.
- We developed a simplified minimally invasive autopsy method, which would be feasible in middle- and low-income countries, and validated it against the complete autopsy, the gold standard, in a series of stillborn babies and neonates who died in Mozambique.

**What did the researchers do and find?:**

- The validity of a minimally invasive autopsy method in determining the cause of death was assessed in 18 stillbirths and 41 neonatal deaths.
- Concordance between the diagnosis obtained with the minimally invasive and the complete diagnostic autopsy was evaluated.
- The minimally invasive autopsy showed a substantial concordance with the complete autopsy in stillborn babies and a moderate concordance in neonates.
- The agreement was particularly high for infectious diseases. Moreover, the specific microorganisms causing death were accurately identified in the minimally invasive autopsy samples.

**What do these findings mean?:**

- The minimally invasive autopsy could be a useful tool for cause of death determination in stillbirths and neonatal deaths.
- These findings may help to accelerate progress towards meeting global health targets by obtaining more accurate information on the causes of death in these age groups, which is essential in guiding the design of new interventions and increasing the effectiveness of those already implemented.

## Introduction

Globally, progress in reducing child mortality has been significantly slower than the target annual rate of decline of 4.4% for children under 5 years established by the previous Millennium Development Goals [1–3]. Peri-neonatal deaths account for nearly half of all under-five child mortality [1,4,5], with nearly 3 million neonatal deaths (within 28 days after birth) and 2.6 million stillbirths (deaths occurring after 28 weeks of gestation) occurring annually. The vast majority of these deaths (99%) take place in low- and middle-income countries, where vital registration systems are inadequate or nonexistent and rarely medically certified [1–5]. This imposes the need to make sophisticated assumptions to establish the etiology in the majority of these deaths [6]. As a consequence, efforts to reduce the burden of peri-neonatal mortality are not always evidence driven, and it is extremely challenging to assess the extent of the progress towards the new Sustainable Development Goals and, thus, to adequately guide policy targeting this age group [7]. Because the causes of most peri-neonatal deaths still remain unknown, rigorous determination of the etiologies of mortality in this age group has been identified as a priority to achieve a significant reduction in this unacceptable burden [8].

In low- and middle-income countries, the 2 conventional sources of information on the causes of mortality, including peri-neonatal deaths, are verbal autopsies and clinical records, both having a significant level of imprecision [9,10]. Verbal autopsies have also been questioned because they are subject to a high degree of misclassification errors, especially for conditions with poor diagnostic specificity such as most causes of peri-neonatal deaths. In fact, although the current WHO verbal autopsy tool adequately identifies newborn deaths and stillbirths by time of death and distinguishes fresh from macerated stillborn babies, this method has many limitations in accurately establishing the cause of death in this group [8]. Clinical records are obtained only from those women who deliver at a health facility or attend it during the postpartum period or for babies who are admitted sick after birth. However, such cases are still a minority in many low- and middle-income countries, as most deliveries take place away from health services. Moreover, for those cases attended at health facilities, clinical diagnostic errors are not infrequent even in well-equipped, tertiary-level hospitals [11].

A reliable ascertainment of the causes of peri-neonatal mortality would require a complete diagnostic autopsy (CDA), a procedure that remains to date the gold standard for cause-of-death determination [12]. Recent studies in high-income countries have highlighted the importance of CDAs in peri-neonatal deaths, as they can correct clinical diagnostic errors and produce information that is additional to the clinical findings in up to 22%–76% of cases [13]. However, performing CDAs in low- and middle-income settings is challenging because of a lack of resources and trained personnel to perform them, the large proportion of deaths occurring outside the health system, and, potentially, cultural and/or religious apprehension about the practice of postmortem procedures, which often makes them unacceptable from the community perspective. In addition to these limitations, restricting the use of the autopsy to in-hospital deaths in these settings introduces an important bias, as the causes of death there may differ from the deaths occurring in the community not reaching the health facilities.

Thus, there is an urgent need to develop simpler and more feasible methods to ascertain accurately the most frequent causes of peri-neonatal death. In recent years, minimally invasive autopsy (MIA) techniques have been developed with this aim [11,14–17]. This method, which involves directed sampling using biopsy needles, provides fluids and tissue samples for pathological and microbiological analyses. The procedure is simple and could be easily conducted by trained technicians. We have recently validated the MIA against the CDA in a series of in-hospital adult deaths from Mozambique (http://journals.plos.org/plosmedicine/article?id=10.1371/journal.pmed.1002173) [18,19] and in a series of pediatric deaths in the same setting [20].

We are presenting here a study on the validity of the MIA to determine the cause of death in a series of stillbirths and neonatal deaths occurring at Maputo Central Hospital (MCH), Mozambique, by comparing the MIA diagnosis with the gold standard diagnosis obtained by the CDA. This study is part of a larger project that also validated the MIA to determine the cause of death in pediatric, maternal, and adult deaths.

## Methods

### Study setting and design

This observational study received the approval of the Clinical Research Ethics Committee of the Hospital Clinic of Barcelona (Spain; approved, File 2013/8677) and the National Bioethics Committee of Mozambique (Mozambique; approved, Ref. 342/CNBS/13). Verbal informed consent was obtained from the relatives of the patients included in the study.

This was a prospective observational study undertaken at the Department of Pathology of MCH, a 1,500-bed government-funded quaternary hospital in southern Mozambique. The complete validation study was carried out between November 2013 and March 2015; details of the study are reported elsewhere [18]. Between April and June 2014, all stillbirths and neonatal deaths fulfilling the inclusion criteria were included in the study; these were (1) a CDA requested by the clinician as part of the medical evaluation; (2) a verbal informed consent to perform the autopsy given by the relatives; (3) a definition of stillbirth (birth of a dead baby with birthweight ≥ 1,000 g, ≥28 completed weeks gestation, or body length ≥ 35cm) [21,22] or neonatal death (a death within 28 days of birth, which includes early neonatal deaths, from 1–7 days, and late neonatal deaths, from 8–28 days) [22,23]; and (4) a less than 24-hour period from death or delivery for neonates and stillborn babies, respectively. Deaths of traumatic origin in neonates were excluded. During this period, up to 2 coupled MIAs and CDAs were conducted per day.

The Strengthening the Reporting of Observational Studies in Epidemiology (STROBE) statement and the prospective analysis plan are included as supplementary information (S1 and S2 Text, respectively). All relevant data are within the paper and its Supporting Information files. Additional data are available upon request, in accordance with the consortium agreement signed by the CaDMIA project partnership. Data use and transfer are monitored by ISGlobal’s Biostatistics and Data Management Unit (contact email: ubioesdm@isglobal.org).

The overall study plan, indicating where the procedures were performed, the investigators involved, and the site and timing of each procedure, has been reported elsewhere [18].

Sample size calculation was done assuming an expected prevalence of infectious diseases as causes of death for all age groups in the overall validation study. A sample size was not estimated to assess the concordance between the MIA and the CDA for each age group and each category of disease. Instead, we included all cases available that met the inclusion criteria during the study period.

### Autopsy procedures

Detailed MIA pathological methods have been reported elsewhere [24,25]. In brief, the procedure included an initial disinfection of the surface of the body, followed by the collection of blood and cerebrospinal fluid, aiming to obtain 10–20 mL of these fluids. Finally, solid organs (liver, lungs, central nervous system, heart, spleen, and kidneys) were punctured using biopsy needles (14–16G). Immediately after the MIA, a second pathologist performed the CDA. In brief, a dissection with macroscopic evaluation of all organs was performed following a standardized protocol for perinatal and pediatric autopsies [26]. Samples from the same viscera collected in the MIA and from any grossly identified lesions were taken during the CDA.

### Histological and microbiological analyses

Two pathologists and 2 microbiologists reviewed and analyzed the samples from the MIA while blinded to any clinical information and to the results of the CDA. All samples collected for histology were stained with hematoxylin and eosin. Histochemical (e.g., PAS stain) and/or immunohistochemical staining (e.g., cytomegalovirus) were used, when needed, to confirm the diagnosis. Microbiological methods have been reported in detail elsewhere [25]. In brief, universal screening that included detection of *Plasmodium falciparum* (by PCR), antibodies against human immunodeficiency virus (HIV)-1/2 and against hepatitis C virus, hepatitis B surface antigen, pathogens (by real-time PCR) included in the TORCH group (*Toxoplasma gondii*, others [includes *Treponema pallidum*, parvovirus B19, and lymphocytic choriomeningitis virus], rubella virus, cytomegalovirus, and herpes virus), and respiratory viruses was performed on all cases, as well as culture of organisms (bacterial and fungal) using samples from the blood, cerebrospinal fluid (CSF), liver, lungs, and central nervous system. In addition, we performed a multiplex real-time PCR test for detection of *Streptococcus pneumoniae*, *Haemophilus influenzae*, *Listeria monocytogenes*, *Neisseria meningitidis*, *Streptococcus agalactiae*, and *Escherichia coli* using a commercial assay (Roche Modular assay). HIV viral load was determined in samples positive for antibodies against HIV. Other microorganisms were tested depending on the pathological findings observed in the MIA-obtained tissues.

Samples from the CDA were analyzed following the same strategy used for the analysis of the MIA samples. The microbiological results of the blood and CSF were also included in the CDA evaluation. The same team of experts evaluated the samples of the CDA without considering the results of the MIA in order to focus on the differences between the 2 methods while minimizing the potential for interobserver variation.

As described elsewhere [18], 2 scales were developed to grade the strength of the evidence of the findings: one was based on the severity of the pathological findings, and the other on the distribution and type of the microorganisms identified. These scales were used in the determination of the certainty of the cause-of-death attribution in both the MIA diagnosis and the CDA diagnosis. For stillborn babies and neonates, the following adaptations were applied to the scales in order to address the particularities of these 2 groups:

1. Body weight was considered as a pathological finding. In stillbirths, weight at birth < the 10th percentile for gestational age and sex (using the INTERGROWTH-21st standards in a stillborn baby [27–29]) was considered as level 2 pathological evidence of fetal growth restriction.
2. Cytomegalovirus and group B streptococcus needed to be always identified in at least 2 different samples to be considered as etiological agents. In the case of cytomegalovirus identification, real-time PCR Ct values ≥ 35 were considered as nonconclusive in the absence of histological evidence.
3. In stillborn babies, strong evidence was given to the identification of pathogens of the TORCH group (except cytomegalovirus), varicella-zoster virus, parvovirus B19, LCMV, group B streptococcus, and *L*. *monocytogenes*.
4. In neonates, strong evidence was given to the identification of *S*. *pneumoniae*, *H*. *influenzae*, *N*. *meningitidis*, *Salmonella* spp., and *Bordetella pertussis*.

### Determination of the cause of death

Once all the analysis of the MIA samples had been completed, a panel composed of a pathologist, a microbiologist, an obstetrician, and a pediatrician with expertise in neonatology evaluated all the data of the MIA and assigned the MIA diagnosis. Investigators involved in the MIA diagnosis were aware of the external macroscopic examination, including anthropometrical measures, and the histological and microbiological results obtained in the MIA sampling but were blind to any other information, including the clinical records and the CDA results. After a washout period (minimum time 3 months, range 3–6 months), the same panel evaluated the data from the CDA and the clinical records and assigned the final diagnosis of the cause of death (CDA diagnosis), which was considered the gold standard. Investigators involved in the CDA diagnosis were aware of the macroscopic (external and internal), histological, and microbiological results and clinical information but were blind to the MIA results. Maternal clinical information was available in 16/18 (89%) stillborn babies and in 24/41 (59%) neonates.

According to the WHO application of the International Classification of Diseases, 10th revision (ICD-10) to deaths during the perinatal period (ICD-PM), a chain of conditions was established following the most probable chronological sequence of events leading to death [28]. When more than 1 severe pathological and/or microbiological diagnosis was identified, the disease most likely causing the death was considered the final diagnosis. Diseases contributing (or possibly contributing) to the death were classified as other diseases or conditions (e.g., prematurity) [28]. Other incidental conditions or concomitant infections not related to the chain of events leading to death were considered as other conditions not likely contributing to the death (e.g., otitis media). Maternal conditions or diseases (e.g., HIV infection) were considered as part of the pathway leading to fetal or infant death.

In each age group, the diseases causing death identified in the CDA or in the MIA were classified into major categories. Thus, in stillbirths the causes of death were classified into 5 categories: (1) fetal growth restriction, (2) infectious diseases, (3) intrauterine hypoxia, (4) intrapartum hypoxia, and (5) nonconclusive. Fetal growth restriction was considered as the cause of death when no other pathological and/or microbiological finding potentially causing death was identified. In neonates, the final cause-of-death diagnoses were classified as (1) infectious diseases, (2) intrapartum complications, (3) preterm complications, (4) congenital malformations, (5) other conditions, and (6) nonconclusive.

### Definitions and statistical methods

The diagnosis of fetal growth restriction was applied to all stillborn babies who were small for gestational age, which was defined as weight at birth < the 10th percentile for gestational age and sex using the INTERGROWTH-21st standards in a stillborn baby [27–29]. Following the ReCoDe classification [30], fetal growth restriction was considered as the direct cause of death in stillbirths, whereas any underlying maternal condition was included in the chain of events. Intrauterine hypoxia and intrapartum hypoxia were defined as the presence of histological signs of hypoxia in the lungs (alveolar capillary dilatation and congestion with interstitial edema, focal hemorrhage, and alveolar ducts distended by squamous epithelial cells) [31] in a macerated or fresh stillborn baby, respectively, in the absence of fetal growth restriction. Intrapartum complications were diagnosed by the presence of signs of hypoxia in the histological examination of the lungs plus a history of intrapartum disorders (e.g., prolonged labor). Prematurity was defined as a gestational age less than 37 weeks, and low birth weight as less than 2.5 kg [27,32]. Maternal HIV infection was defined as the presence of HIV antibodies in fetal or neonatal blood.

The concordance between the categories of diseases obtained by the MIA and the CDA (gold standard) diagnoses was assessed by the Kappa statistic, and it was interpreted as suggested by Landis and Koch [33]. The diagnostic accuracy of the MIA to identify the categories established by the CDA diagnosis was evaluated as sensitivity, specificity, positive and negative predictive values, and the total percentage of cases correctly classified.

## Results

### Characteristics of stillbirths and neonatal deaths

Coupled MIA and CDA procedures were performed in 18 stillborn babies and 41 neonates. Twelve (67%) stillborn babies were macerated (3 grade I, 4 grade II, and 5 grade III). The median gestational age of the stillborn babies was 36 weeks (range 30 to 40). The range of weights at birth varied between 1,050 and 3,500 g. Twenty-nine (71%) neonates died during the early neonatal period, and 12 (29%) in the late neonatal period. Seven (39%) stillborn babies and 6 (15%) neonates tested positive for antibodies against HIV (all of them HIV-1). In addition, HIV RNA was detected by PCR in 1 neonate. In 3 (17%) stillborn babies, there was a history of malaria during pregnancy, while in 5 (12%) neonates *P*. *falciparum* was detected by PCR in blood collected onto filter paper. The sex of the babies, neonatal age at death, and the number of preterm and low-birth-weight babies are shown in Table 1.

**Table 1:** Characteristics of stillbirths and neonatal deaths.

	Stillbirths *n* = 18 (%)	Neonates *n* = 41 (%)
**Sex**		
Female	6 (33)	14 (34)
Male	12 (67)	27 (66)
**Neonatal age at death**		
Early neonatal period	N/A	29 (71)
Late neonatal period	N/A	12 (29)
**Preterm (<37 weeks of gestation)** ^(1)^	11 (61)	18 (46)
**Low birth weight (<2,500 g at birth)**	N/A	25 (61)
**Small for gestational age (weight at birth < 10th percentile for gestational age and sex)** ^(2)^	7 (39)	12 (35)
**Death timing according to delivery**		
Antepartum	12 (67)	N/A
Intrapartum	6 (33)	N/A

N/A, not applicable.

^(1)^ Information not available in 2 neonates;

^(2)^ information not available in 7 neonates.

Less than 10 mL of blood was obtained in 1 (6%) stillbirth and in 3 (7%) neonates. In all these cases, neither antibodies against HIV-1/2 nor viral load could be determined because of insufficient plasma volume. All the mothers of the study participants were known to be alive except in 3 cases for which this information was not available.

### Concordance between the MIA diagnosis and the CDA diagnosis in stillborn babies

A cause of death was identified in the CDA (gold standard) in 16 out of 18 (89%) stillbirths. Fetal growth restriction accounted for 7 (39%), infectious diseases for 4 (22%), intrauterine hypoxia for 2 (11%), and intrapartum hypoxia for 3 (17%) of the deaths (Table 2). A MIA diagnosis of cause of death was identified in 15 out of 18 (83%) stillborn babies. Table 2 shows the concordance between the MIA and the CDA diagnoses of stillborn babies, grouped according to the major disease categories. The MIA categorization of disease showed a substantial concordance with the gold standard (Kappa = 0.78, 95% CI [0.56–0.99]) and agreed in 15/18 (83%) of the cases.

**Table 2:** Concordance of the categorization of the causes of death established by the complete diagnostic autopsy (CDA, gold standard) and the minimally invasive autopsy (MIA) in stillbirths.

MIA diagnosis	CDA diagnosis (gold standard)					
	Fetal growth restriction	Infectious disease	Intrauterine hypoxia	Intrapartum hypoxia	Nonconclusive	Total
**Fetal growth restriction**	**7**	0	0	0	0	7 (39%)
**Infectious diseases**	0	**3**	0	1	0	4 (22%)
**Intrauterine hypoxia**	0	1	**2**	0	0	3 (17%)
**Intrapartum hypoxia**	0	0	0	**1**	0	1 (6%)
**Nonconclusive**	0	0	0	1	**2**	3 (17%)
**Total**	7 (39%)	4 (22%)	2 (11%)	3 (17%)	2 (11%)	18

Kappa value = 0.78, 95% CI (0.56–0.99).

S1 Table shows the diagnoses obtained in the CDA (gold standard) and the MIA for each case. In the 7 stillbirths with fetal growth restriction, the information registered in the obstetric clinical record identified a maternal condition likely to be associated with the intrauterine growth disorder as follows: a clinical history of eclampsia/preeclampsia was identified in 3 cases (one of the women also had a record of malaria infection and anemia during pregnancy, and another was also positive for HIV), maternal anemia alone was identified in 1 case, maternal HIV infection alone was detected in 2 cases, and 1 case was a multiple pregnancy (triplets). Maternal HIV infection was reported in the obstetric history of 7 stillborn babies; this infection was identified in the MIA through detection of HIV antibodies in fetal blood, with those making the identification blinded to the obstetric clinical record (S1 Table). Group B streptococcus (GBS) infection was identified in 3 stillborn babies in both the MIA and the CDA, but only in 2 of them was the GBS infection considered the cause of death in the CDA (in 1 of them, maternal HIV infection was detected in the MIA and reported in the obstetric record).

### Concordance between the MIA diagnosis and the CDA diagnosis in neonates

The CDA identified a diagnosis in all cases (100%). Of them, 27 (66%) were diagnosed with infectious diseases, 5 (12%) had preterm complications, 4 (10%) had congenital malformations, 3 (7%) were classified as having birth asphyxia due to intrapartum complications, and 2 (5%) were classified as having other conditions (Table 3). Neonatal sepsis accounted for most of the infectious diseases (21/27, 78%). A MIA diagnosis was identified in 35/41 (85%) of neonatal deaths. S2 Table shows the diagnoses obtained by the CDA and the MIA for each case. Four (10%) neonates had GBS infection, and in 3 of them it was considered the cause of death in the CDA.

**Table 3:** Concordance of the categorization of the cause of death established by the complete diagnostic autopsy (CDA, gold standard) and the minimally invasive (MIA) diagnosis in neonates.

MIA diagnosis	CDA diagnosis (gold standard)						
	Infectious diseases	Preterm complications	Congenital malformations	Intrapartum complications	Other conditions	Nonconclusive	Total
**Infectious diseases**	**23**	1	0	3	1	0	28 (68%)
**Preterm complications**	1	**3**	0	0	0	0	4 (10%)
**Congenital malformations**	0	0	**2**	0	0	0	2 (5%)
**Intrapartum complications**	0	0	0	**0**	0	0	0 (0%)
**Other conditions**	0	0	1	0	**0**	0	1 (2%)
**Nonconclusive**	3	1	1	0	1	**0**	6(15%)
**Total**	27 (66%)	5 (12%)	4 (10%)	3 (7%)	2 (5%)	0 (0%)	41

Kappa = 0.40, 95% CI (0.18–0.63)

The categorization of cause of death according to the MIA diagnosis showed a moderate concordance with the categorization reached with the CDA (Kappa = 0.40, 95% CI [0.18–0.63]) and agreed in 28/41 (68%) of the neonatal deaths (Table 3). The concordance was higher for infectious diseases (23/27; 85%) and preterm complications (3/5; 60%) than for congenital malformations (2/4; 50%). None of the cases diagnosed in the CDA as intrapartum complications or other diseases were identified in the MIA.

Table 4 shows the list of etiologic agents identified in the CDA and the MIA and the number of cases in which the same etiologic agent was identified; for example, in the CDA, neonatal sepsis due to mixed infections of *Enterobacteriaceae* was diagnosed in 5 cases, while in the MIA this was diagnosed in 3 cases, and in 2 of these cases, the same etiologic agent was detected in both the MIA and CDA. The specific microorganisms causing death were identified in the CDA in 26/27 (96%) of the infectious deaths (Table 4). The MIA identified the same microorganism in 17/26 (65%) of the cases.

**Table 4:** List of microorganisms identified as the cause of death in the complete diagnostic autopsy (CDA, gold standard) and in the minimally invasive autopsy (MIA) in neonates.

Microorganisms identified by infection group	CDA (*n*)	MIA (*n*)	CDA and MIA (*n*)
**Neonatal sepsis**	**21**	**21**	**14**
Enterobacteriaceae*	5	3	2
*E*. *coli*	3	4	3
Group B streptococcus (GBS)	3	0	0
*Klebsiella pneumoniae*	3	5	2
*Salmonella* spp.	3	3	3
*S*. *pneumoniae*	2	2	2
*Acinetobacter baumanii*	1	1	1
Mixed gram-negative bacteria**	0	1	0
No agent	1	2	1
**TORCH syndrome**†	**3**	**2**	**2**
Cytomegalovirus	1	1	1
Herpes simplex virus type 2	2	1	1
**Other infections**	**3**	**5**	**1**
*Enterobacteriaceae* (pneumonia)*	1	0	0
*E*. *coli* (pneumonia)	0	1	0
Parainfluenza virus (pneumonia)	1	0	0
Respiratory syncytial virus (pneumonia)	0	2	0
*S*. *pneumoniae* (meningitis)	1	1	1
No agent (pneumonia)	0	1	0
**Total**	**27**	**28**	**17**

* Mixed infection of at least 2 of these enterobacteria: *Enterobacter cloacae*, *E*. *coli*, or *K*. *pneumoniae*.

** Gram-negative bacteria included the following: *A*. *baumanii* + *K*. *pneumoniae* + *E*. *coli*.

^†^ TORCH syndrome includes infections by *T*. *gondii*, *T*. *pallidum*, parvovirus B19, lymphocytic choriomeningitis virus, herpes virus, cytomegalovirus, and rubella virus.

### Diagnostic accuracy of the MIA

Table 5 shows the sensitivity, specificity, positive predictive value, negative predictive value, and accuracy of the MIA for the different diagnostic categories in the CDA. Although the numbers are small, there was a high percentage of accuracy of the MIA for the different diagnostic categories both in stillbirths and neonatal deaths (>75%).

**Table 5:** Sensitivity, specificity, positive and negative predictive value (PPV and NPV), and accuracy of the minimally invasive autopsy (MIA) for the different diagnostic categories in stillbirths and neonates. Figures are percentages and 95% confidence intervals.

Cause of death category	*n*	Sensitivity	Specificity	PPV	NPV	Accuracy
**Stillbirths**						
**Fetal growth restriction**	7	100	100	100	100	100
		(59–100)	(72–100)	(59–100)	(72–100)	(81–100)
**Infectious diseases**	4	75	93	75	93	89
		(19–99)	(66–100)	(19–99)	(66–100)	(65–99)
**Intrauterine hypoxia**	2	100	94	67	100	94
		(16–100)	(70–100)	(9–99)	(78–100)	(73–100)
**Intrapartum hypoxia**	3	33	100	100	88	89
		(1–91)	(78–100)	(2–100)	(64–99)	(65–99)
**Nonconclusive**	2	100	94	67	100	94
		(16–100)	(70–100)	(9–99)	(78–100)	(73–100)
**Neonates**						
**Infectious disease**	27	85	64	82	69	78
		(66–96)	(35–87)	(63–94)	(39–91)	(62–89)
**Preterm complications**	5	60	97	75	95	93
		(15–95)	(85–100)	(19–99)	(82–99)	(80–98)
**Congenital malformations**	4	50	100	100	95	95
		(7–93)	(91–100)	(16–100)	(83–99)	(83–99)
**Intrapartum complications**	3	0	100	N/A	93	93
		(0–71)	(91–100)		(80–98)	(80–98)
**Other disease**	2	0	97	0	95	93
		(0–84)	(87–100)	(0–98)	(83–99)	(80–98)
**Nonconclusive**	0	N/A	85	0	100	85
			(71–94)	(0–46)	(90–100)	(71–94)

N/A, not applicable.

### Other significant conditions contributing to death in stillborn babies and neonates

No other significant fetal condition contributing to death was detected in the series of stillbirths. A maternal condition contributing to death was identified in 14 out of 18 (78%) stillborn babies (S1 Table). Maternal HIV and preeclampsia (7/14, 50%, and 4/14, 29%, respectively) were the most frequent underlying maternal conditions. The other 3 cases were 1 maternal pneumonia, 1 multiple pregnancy (triplets), and 1 umbilical cord prolapse. Maternal malaria was identified as other significant condition leading to death in 3/18 cases (17%).

In neonates, other significant conditions contributing to death were identified in 27 out of 41 (66%) cases (S2 Table). The most frequent condition was prematurity, observed in 17/27 (63%) of the neonates. The remaining conditions consisted of 4 intrapartum complications, 2 cases of omphalitis, 1 case of omphalocele, 1 case of meningitis, 1 case of intra-uterine HIV infection, and 1 case of necrotizing enterocolitis. In 20/27 cases (74%), these contributory conditions were identified in the MIA procedure. Maternal HIV infection and congenital malaria (5/41,12%, and 1/41, 2%, respectively) were identified as maternal conditions contributing to death (S2 Table).

## Discussion

To our knowledge, this is the first study to evaluate the validity of a minimally invasive postmortem procedure in stillbirths and neonatal deaths by comparing the results obtained with this approach with those achieved in the CDA, the gold standard for cause-of-death determination. The results show that the MIA diagnosis has a high concordance and percentage of agreement with the CDA diagnosis, and thus, the MIA could be a useful tool for cause of death determination in both stillborn babies and neonates. The MIA may contribute to obtaining more accurate information on the causes of mortality in these particular groups, which is essential in guiding the target and design of new interventions, as well as increasing the effectiveness of those already implemented [8].

Fetal growth restriction and infectious diseases were the most frequent causes of death in stillbirths, followed by intrauterine and intrapartum hypoxia. In 7 stillborn babies, the mother had HIV infection, which could be ascertained in the MIA through detection of HIV antibodies in the fetal blood. Thus, not only the cause of death but also some maternal medical conditions contributing to death could be identified in the MIA even in the absence of any clinical information from the obstetric record. Although the obstetric history is important in determining the cause of death, mainly in stillbirths, we were able to ascertain the cause of death in the MIA in the majority of stillbirths and neonates without making use of the obstetric clinical record. This is of relevance since in many high-burden settings the maternal record is not available because the delivery usually takes place outside the health facility or, if it exists, is often incomplete. However, some relevant maternal conditions known to be the underlying cause of fetal deaths, particularly those causing fetal growth restriction, such as preeclampsia/eclampsia and maternal anemia, will not be captured unless specific clinical data are obtained. In addition, relevant information could be also indirectly retrieved by including a careful placental examination as part of the MIA.

In 2 stillborn babies in whom the cause of death was related to intrauterine hypoxia both in the MIA and the CDA, only maternal HIV antibodies were detected. In addition, maternal HIV infection was observed in another 2 growth-restricted cases. Thus, maternal HIV infection was likely to be indirectly responsible for the death of more than a third of the stillbirths in this series. With the limitation intrinsic to small numbers, this figure is much higher than the recent estimation of the attribution of stillbirths to HIV in sub-Saharan Africa (0.7%) [8]. Although antiretroviral drugs and programs of prevention of mother-to-child transmission have been scaled up in the last decade, the prevalence of HIV infection remains high in Mozambique [34]. From these findings, it is clear that more efforts are needed to control this infection, which would have a significant impact in reducing a considerable proportion of preventable fetal deaths. In the same systematic review, it has been estimated that maternal malaria is associated with 20% of stillbirths in sub-Saharan Africa [8]. In our series, a history of malaria infection during pregnancy was reported in 3 stillborn babies (17%) whose final causes of death were determined as intrauterine hypoxia (2) and fetal growth restriction (1). Larger series of postmortem studies in rural areas with higher malaria endemicity than urban Maputo are needed to conclusively establish the impact of malaria in pregnancy as a cause of fetal deaths.

Infectious diseases were the second most important cause of death in stillbirths in this study, which is in agreement with previously reported estimations [8]. There were 2 cases of GBS infection, 1 case of chorioamnionitis, and 1 case of a disseminated *E*. *coli* infection. Though the numbers are small, the proportion (16%) of GBS-related stillbirths observed in this study is higher than that reported in a recent systematic review [35]. In 2 cases, the CDA and the MIA were concordant, while in the third case GBS infection was only considered as being the cause of death in the MIA. Two of these cases were observed in macerated stillbirths, indicating that death was likely to have occurred antepartum. Although GBS infection seems to be a more frequent cause of neonatal sepsis at least in high-income countries, GBS-associated stillbirths are not infrequent, and their prevention would require a different strategy than the currently recommended intrapartum antibiotic prophylaxis for colonized or at-risk women [36].

In stillborn babies, an external examination of the body determining whether it is fresh or macerated provides information on timing of demise, which is critical to classify the death as occurring ante- or intrapartum. In contrast with high-income countries, in low- and middle-income regions the majority of stillbirths are intrapartum [8,10,36]. In this study, more than a third of the stillbirths were intrapartum defined by the absence of skin maceration. However, it cannot be ruled out that in a number of macerated stillborn the death also occurred during labor if there was a delay in the access to adequate intrapartum care. Therefore, in contexts of scarce resources and limited access to high-quality care, it is difficult to get a precise estimate of the intrapartum stillbirth rate. This limits the use of this indicator as a marker of fetal death avoidable through improved care during labor, precisely in settings where it could be more helpful. Similarly, a challenge in resource-poor settings is distinguishing between intrapartum stillbirths and very early neonatal deaths. However, to our knowledge it is not possible to differentiate between the 2 conditions in the histological examination, and it was not part of the study to make this distinction; thus, we had to rely on the clinical judgment and the macroscopic examination.

In neonates, infectious diseases accounted for the majority of deaths (66%), followed by preterm complications, congenital malformations, and intrapartum-related complications. Most of the infections were sepsis, and congenital infections accounted for 3 cases of TORCH (2 herpesvirus type 2 and 1 cytomegalovirus), 2 cases of pneumonia, and 1 case of meningitis. As for stillbirths, GBS infection seemed to be an important cause of death in this group, either as the final cause (3 cases) or as a coinfection with CMV and HIV (1 case). Only in the latter case was there concordance between the MIA and the CDA. In neonates, the concordance between the CDA and the MIA was moderate, being the highest percentage of concordance (23/27, 85%) for infectious diseases. Of note, all intrapartum complications in the CDA examination were missed in the MIA. It is important to remember that as it is routine practice, in this study the clinical data, which include the obstetric record, were part of the information evaluated for cause-of-death attribution in the CDA, but not in the MIA. Therefore, the CDA alone without the clinical information would have probably missed these cases as well; on the other hand, inclusion of this information in the MIA cause-of-death attribution would have probably captured these cases. It is also important to notice that the microorganism could be identified in most deaths due to infectious diseases in the CDA but also in the MIA. This information is critical to improve the specificity and, therefore, the cost-effectiveness of targeted interventions and may also help to reduce the burden of antibiotic resistance in relation to the cause of death, a major global public health problem [37]. Although preterm labor is usually mentioned as a cause of neonatal death, this is usually a midfactor in the causal pathway. In this study, although prematurity was identified in over 40% of neonatal deaths, it was considered generally an underlying condition and not the final cause of death. Importantly, we could determine factors that may be causing prematurity (most of them infections), which are the ones that should be tackled by specific interventions. The proportion of male babies was higher than that of female babies both for stillbirths and neonatal deaths. The excess risk of death in male fetuses and newborns has been reported [38]. Although the reasons are unclear, it is suggested to be due to an increased risk of preterm birth and fetal growth restriction and X-linked congenital abnormalities, as well as to a higher incidence of placental vascular conditions [38].

The objective of this validation study was not to describe the causes of stillbirths and neonatal deaths. Thus, although the main causes of mortality identified in this study are similar to those previously reported so far, a conclusive and comprehensive description would need larger studies in different settings. Similarly, because of small numbers, the precision in the estimates of proportions is limited. Consequently, the sensitivity, specificity, positive predictive value, negative predictive value, and the percentage of cases correctly classified may vary with slight modifications in the classification of a few cases. In addition, a limitation of this study in the case of stillbirths is the lack of inclusion of early fetal deaths in the validation of the MIA against the CDA. This should be considered in future validations of this methodology. It is noteworthy that clinical findings, including maternal information, were essential to reach the final cause of death in a number of CDA diagnoses (6 cases). Thus, it is likely that the inclusion of clinical and obstetric information or, in its absence, data provided by the verbal autopsy could significantly improve the results of the MIA in these age groups. We did not include placental histologic/microbiologic examination in this MIA protocol, which may have enriched the ascertainment of diagnosis in some cases, since some maternal conditions such as preeclampsia and chorioamnionitis can be recognized in the placenta, while intrauterine hypoxia could be caused by placental abnormalities. Fetal growth restriction in stillborn babies was estimated using weight and gestational age. The latter was the only parameter obtained from the clinical record because it was necessary to classify the stillbirth. In the absence of this information, other anthropometric measurements such as foot length could be used [8,26,39]. In both stillborn babies and neonates, the MIA showed a high concordance with the CDA for infectious diseases but performed poorly in detecting congenital malformations. The blind sampling scheme of the MIA protocol does not allow macroscopic observation of the internal organs, but the observation of the entire body prior to the procedure or pictures of it could help to identify some external malformations.

It has been stated that better data are critical to accelerate progress towards the target of 12 or fewer stillbirths per 1,000 births and 12 or less neonatal deaths per 1,000 live births by 2030 [5,8,40]. Better statistics on the number of these deaths and, even more importantly, accurate information on the causes of mortality are essential for health planning, priority setting, designing effective health programs, and evaluating their impact, besides playing a role in the accountability of implemented interventions. The MIA approach may contribute to reducing peri-neonatal deaths from the more than 5 million annual deaths, by providing a tool that can be utilized by trained nurses and midwives in rural areas, where at least 60% of stillbirths and neonatal deaths occur, to establish the cause of death.

### Conclusion

Most peri- and neonatal deaths occur in countries with very scarce resources to undertake more complex postmortem examinations, relying on verbal autopsies (VAs) to determine the cause of death in particular for births outside health facilities. However, VAs have many limitations in accurately establishing the cause of death in these groups [41]. The MIA approach, although still imperfect as compared to the gold standard (CDA plus clinical information), may provide unique information for health prioritization, which currently relies just on both indirect and inaccurate estimations and assumptions on the causes of mortality of stillborn babies and neonates in most parts of the world. This approach should also help at saving global health funds by directing expenditures to the most-effective health programs.

## Supporting information

S1 Table Gold standard and minimally invasive autopsy cause-of-death results of each stillborn baby. (DOCX)

S2 Table Gold standard and minimally invasive autopsy cause-of-death results of each neonatal death. (DOCX)

S1 Text STROBE checklist for the observational study developed in Mozambique to validate the minimally invasive autopsy for cause-of-death determination in stillborn babies and neonates. (DOCX)

S2 Text Prospective analysis plan on pathological and microbiological procedures used during the study. (DOCX)

## Abbreviations

CDA: complete diagnostic autopsy
CSF: cerebrospinal fluid
GBS: group B streptococcus
ICD-10: International Classification of Diseases, 10th revision
ICD-PM: the WHO application of ICD-10 to deaths during the perinatal period
MCH: Maputo Central Hospital
MIA: minimally invasive autopsy
N/A: not applicable
NPV: negative predictive value
OR: odds ratio
PPV: positive predictive value
STROBE: Strengthening the Reporting of Observational Studies in Epidemiology
TORCH: *Toxoplasma gondii*, others (includes *Treponema pallidum*, parvovirus B19, and lymphocytic choriomeningitis virus), rubella virus, cytomegalovirus, and herpes virus
VA: verbal autopsy
