# Supplementary material for: Validity of a minimally invasive autopsy for cause of death determination in stillborn babies and neonates in Mozambique: An observational study
Source: PLoS Med. 2017 Jun 20;14(6):e1002318. doi: 10.1371/journal.pmed.1002318 (PMC5478138; doi:10.1371/journal.pmed.1002318)
Supplement: S1 Table — (DOCX) [file pmed.1002318.s001.docx]

| **Cause of death category** | **Case Num.** | **Grade of maceration** | **Gestational age** | **Weight (g)** | **CDA** | | **MIA** | |
| --- | --- | --- | --- | --- | --- | --- | --- | --- |
|  |  |  |  |  | **Final diagnosis** | **Maternal significant condition ^a^** | **Putative diagnosis** | **Maternal significant condition** |
| **Fetal growth restriction (FGR)** | 1 | NA (Fresh) | 31 | 1100 ^a^ | FGR | Eclampsia | FGR | - |
|  | 2 | II | 33 | 1500 ^a^ | FGR | Eclampsia. Maternal malaria and anemia | FGR | - |
|  | 3 | III | 40 | 2300 ^a^ | FGR | Preeclampsia. Maternal HIV infection | FGR | Maternal HIV infection |
|  | 4 | II | 34 | 1400 ^a^ | FGR | Maternal HIV infection | FGR | Maternal HIV infection |
|  | 5 | III | 40 | 2200 ^a^ | FGR | Maternal HIV infection | FGR | Maternal HIV infection |
|  | 6 | NA (Fresh) | 36 | 2000 ^a^ | FGR | Maternal anemia | FGR | - |
|  | 7 | III | 30 | 1050 ^a^ | FGR | Triple pregnancy | FGR | - |
| **Infectious disease** | 8 | II | 36 | 2600 | GBS infection | Maternal HIV infection | GBS infection | Maternal HIV infection |
|  | 9 | III | 37 | 3450 | GBS infection | No information | GBS infection | - |
|  | 10 | NA (Fresh) | 30 | 1600 | Suggestive of chorioamnionitis | Chorioamnionitis | Gram negative bacterial infection | - |
|  | 11 | III | 40 | 3200 | Disseminated infection (*E. coli*) | Maternal HIV infection | Intrauterine hypoxia | Maternal HIV infection |
| **Intrapartum hypoxia** | 12 | NA (Fresh) | 40 | 3600 | Intrapartum hypoxia | Umbilical cord prolapse | Intrapartum hypoxia | - |
|  | 13 | NA (Fresh) | 35 | 2100 | Intrapartum hypoxia ^c^ | Eclampsia | GBS infection | - |
|  | 14 | NA (Fresh) | 36 | 2890 | Intrapartum hypoxia | Eclampsia. Maternal malaria infection | Non conclusive | - |
| **Intrauterine hypoxia** | 15 | I | 34 | 2300 | Intrauterine hypoxia | Maternal HIV infection. Maternal malaria infection | Intrauterine hypoxia | Maternal HIV infection |
|  | 16 | I | 34 | 1950 | Intrauterine hypoxia | Maternal HIV infection | Intrauterine hypoxia | Maternal HIV infection |
| **Non conclusive** | 17 | II | 40 | 2700 | Non conclusive | No information | Non conclusive | - |
|  | 18 | I | 37 | 2300 | Non conclusive | No information | Non conclusive | - |

FGR: Fetal Growth Restriction; GBS: Group B Streptococcus; NA: Not applicable

^a^ Weight at birth <10^th^ percentile for gestational age and sex using the INTERGROWTH-21^th^ standards in a stillborn baby [26–28].

^b^ All information except the maternal HIV status was obtained from the obstetric clinical record.

^c^ A GBS infection was also detected in the CDA.
