# Supplementary material for: Validity of a minimally invasive autopsy for cause of death determination in stillborn babies and neonates in Mozambique: An observational study
Source: PLoS Med. 2017 Jun 20;14(6):e1002318. doi: 10.1371/journal.pmed.1002318 (PMC5478138; doi:10.1371/journal.pmed.1002318)
Supplement: S2 Table — (DOCX) [file pmed.1002318.s002.docx]

|  | **CDA** | | | | **MIA** | | | |
| --- | --- | --- | --- | --- | --- | --- | --- | --- |
| **Case** | **CDA diagnosis** | **Other neonatal significant conditions likely contributing to death** | **Other neonatal conditions** | **Maternal significant condition** | **MIA diagnosis** | **Other neonatal significant conditions likely contributing to death** | **Other neonatal conditions** | **Maternal significant conditions** |
| **Infectious diseases** | | | | | | | | |
| 1 | Neonatal sepsis (*E. coli*)* | Prematurity |  | Premature membrane rupture | Neonatal sepsis (*E. coli*)* | Prematurity |  |  |
| 2 | Neonatal sepsis (*E. coli*)* | Meningitis | Acute medial otitis | Maternal HIV infection | Neonatal sepsis (*E. coli*)* |  |  | Maternal HIV infection |
| 3 | Neonatal sepsis (*E. coli*)* | Prematurity |  | Premature membrane rupture | Neonatal sepsis (*E. coli*)* | Prematurity |  |  |
| 4 | Neonatal sepsis (*Enterobacteriaceae*)** | Prematurity |  | Maternal HIV infection | Neonatal sepsis (*Enterobacteriaceae*)** | Prematurity |  | Maternal HIV infection |
| 5 | Neonatal sepsis (*Enterobacteriaceae*)** | Prematurity |  |  | Neonatal sepsis (*Enterobacteriaceae*)** | Prematurity |  |  |
| 6 | Neonatal sepsis (*Enterobacteriaceae*)** |  | Rhinovirus | Premature membrane rupture. Maternal HIV infection | Neonatal sepsis (Gram-negative bacteria)*** |  | Rhinovirus | Maternal HIV infection |
| 7 | Neonatal sepsis (*Enterobacteriaceae*)** | Prematurity |  |  | Non-conclusive | Prematurity |  |  |
| 8 | Neonatal sepsis (*Enterobacteriaceae*)** | Prematurity | Congenital malaria | Premature membrane rupture | Neonatal sepsis (No agent) | Prematurity | Congenital malaria |  |
| 9 | Neonatal sepsis (*S. pneumoniae*) |  | *P. falciparum* (not quantifiable) |  | Neonatal sepsis (*S. pneumoniae*) |  | *P. falciparum* (not quantifiable) |  |
| 10 | Neonato sepsis (Group B Streptococcus) | Omphalitis |  |  | Neonatal sepsis (*K. pneumoniae*) | Omphalitis |  |  |
| 11 | Neonato sepsis (Group B Streptococcus) | Necrotizing enterocolitis | RSV |  | Pneumonia (RSV) |  |  |  |
| 12 | Neonato sepsis (Group B Streptococcus) |  |  | Maternal severe anemia | Non conclusive |  |  |  |
| 13 | Neonatal sepsis (*S. pneumoniae*) |  |  | Premature membrane rupture | Neonatal sepsis (*S. pneumoniae*) |  |  |  |
| 14 | Neonatal sepsis *(K. pneumoniae)** | Hypoxic - Ischemic Encephalopathy | *T. gondii, P. falciparum* (not quantifiable) | Maternal HIV infection | Neonatal sepsis *(K. pneumoniae)** |  | *P. falciparum* (not quantifiable), *T. gondii* | Maternal HIV infection |
| 15 | Neonatal sepsis *(K. pneumoniae)** |  |  |  | Neonatal sepsis *(K. pneumoniae)** |  |  |  |
| 16 | Neonatal sepsis *(K. pneumoniae)** |  |  |  | Non-conclusive |  |  |  |
| 17 | Neonatal sepsis (*A. baumannii*)* | Prematurity | *P. falciparum* (not quantifiable) | Premature membrane rupture. maternal preeclampsia | Neonatal sepsis (*A baumanii*)* | Prematurity | *P. falciparum* (not quantifiable) |  |
| 18 | Neonatal sepsis (No agent) | Omphalitis |  |  | Neonatal sepsis (No agent) |  |  |  |
| 19 | Neonatal sepsis (*Salmonella spp*) | Omphalocele | RSV | Maternal HIV infection | Neonatal sepsis (*Salmonella spp)* | Omphalocele |  | Maternal HIV infection |
| 20 | Neonatal sepsis (*Salmonella spp*) |  |  |  | Neonatal sepsis (*Salmonella spp*) |  |  |  |
| 21 | Neonatal sepsis (*Salmonella spp*) | Prematurity |  | Premature membrane rupture | Neonatal sepsis (*Salmonella spp*) | Prematurity |  |  |
| 22 | Congenital infection (CMV) | HIV | GBS | Premature membrane rupture | Congenital infection (CMV) | HIV | GBS |  |
| 23 | Congenital herpes (HSV-2) | Prematurity |  | Premature membrane rupture. Maternal HIV infection | Congenital herpes (HSV-2) | Prematurity |  | Maternal HIV infection |
| 24 | Congenital herpes (HSV-2) | Prematurity | *P. falciparum* (not quantifiable) | Premature membrane rupture | Neonatal sepsis (*K. pneumoniae*)* | Prematurity | *P. falciparum* (not quantifiable) |  |
| 25 | Pneumonia (*Enterobacteriaceae*)** | Prematurity |  | Multiple pregnancy. Premature membrane rupture | Pneumonia (*E. coli)* | Prematurity |  |  |
| 26 | Pneumonia (Parainfluenza virus) | Prematurity | Cerebral hemorrhage | Premature membrane rupture | Hyaline membrane disease (HMD) | Prematurity |  |  |
| 27 | Meningitis (*S. pneumoniae*) |  |  |  | Meningitis (*S. pneumoniae*) |  |  |  |
| **Preterm complications** | | | | | | | | |
| 28 | Hyaline membrane disease (HMD) | Prematurity | *P. falciparum* (not quantifiable) |  | Hyaline membrane disease (HMD) | Prematurity | *P. falciparum* (not quantifiable) |  |
| 29 | Hyaline membrane disease (HMD) | Prematurity | *K. pneumoniae*. *P. falciparum* (not quantifiable) | Multiple pregnancy | Hyaline membrane disease (HMD) | Prematurity | *P. falciparum* (not quantifiable) |  |
| 30 | Hyaline membrane disease (HMD) | Prematurity | *S. pneumoniae* | Premature membrane rupture | Neonatal sepsis (*Enterobacteriaceae*)** | Prematurity with HMD |  |  |
| 31 | Hyaline membrane disease (HMD) | Prematurity |  | Multiple pregnancy. Premature membrane rupture | Hyaline membrane disease (HMD) | Prematurity |  |  |
| 32 | Pulmonary hemorrhage | Prematurity |  | Preeclampsia. premature membrane rupture | Non-conclusive |  |  |  |
| **Congenital anomalies** | | | | | | | | |
| 33 | Anencephaly |  |  |  | Anencephaly |  |  |  |
| 34 | Lissencephaly |  |  |  | Non-conclusive |  |  |  |
| 35 | Limb-body wall syndrome/complex |  | *Enterobacteriaceae* |  | Limb-body wall syndrome/complex |  | *Enterobacteriaceae* |  |
| 36 | Congenital cardiopathy (single ventricle heart defect) |  |  |  | Pulmonary hemorrhage |  |  |  |
| **Intrapartum complications** | | | | | | | | |
| 37 | Pulmonary hemorrhage | Prolonged labor | *K. pneumoniae* |  | Neonatal sepsis *(K. pneumoniae)** |  | Adenovirus |  |
| 38 | Birth asphyxia | Fetal distress (meconium) | RSV, CNS hemorrhage |  | Pneumonia (RSV) |  |  |  |
| 39 | Birth asphyxia | Hypoxic - Ischemic encephalopathy | *E. coli* |  | Neonatal sepsis (*E. coli*)* |  |  |  |
| **Other conditions** | | | | | | | | |
| 40 | Intestinal occlusion |  |  |  | Pneumonia (no agent) |  |  |  |
| 41 | Kernicterus |  |  |  | Non-conclusive |  |  |  |

GBS: Group B Streptococcus; HSV-2: Herpes virus simplex type 2; RSV: Respiratory syncytial virus.

* Because their high detection rate, *A. baumannii*, *E. coli* and *K. pneumoniae* were confirmed by culture and PCR methods in at least 2 different samples of the same case before being considered in the diagnosis of the cause of death.

** “*Enterobacteriaceae”* reports a mixed infection of at least two of these enterobacteria: *Enterobacter cloacae*, *Escherichia coli*, or *Klebsiella pneumoniae*

*** Mixed infection of: *A baumanii* + *K. pneumoniae* + *E. coli*
