# Supplementary material for: Validity of a minimally invasive autopsy for cause of death determination in stillborn babies and neonates in Mozambique: An observational study
Source: PLoS Med. 2017 Jun 20;14(6):e1002318. doi: 10.1371/journal.pmed.1002318 (PMC5478138; doi:10.1371/journal.pmed.1002318)
Supplement: S1 Text — (DOCX) [file pmed.1002318.s003.docx]

**Supplementary information - STROBE checklist**

|  |  | | | Recommendation |  | | | | |
| --- | --- | --- | --- | --- | --- | --- | --- | --- | --- |
| **Title and abstract** | | | | | | | | | |
|  | 1 | | | (*a*) Indicate the study’s design with a commonly used term in the title or the abstract | OK: Title; Abstract [Methods and Findings, paragraph 1] | | | | |
|  |  |  |  | (*b*) Provide in the abstract an informative and balanced summary of what was done and what was found | OK: Abstract [Methods and Findings, paragraphs 1-2] | | | | |
| Introduction | | | | |  | | | | |
| Background/rationale | 2 | | | Explain the scientific background and rationale for the investigation being reported | OK: Abstract [Background, paragraph 1]; Author summary [Why Was This Study Done?, bullet points 1-6]; Introduction [paragraphs 1-3] | | | | |
| Objectives | 3 | | | State specific objectives, including any pre-specified hypotheses | OK: Introduction [paragraph 5] | | | | |
| Methods | | | | |  | | | | |
| Study design | 4 | | | Present key elements of study design early in the paper | OK: Abstract [Methods and findings, paragraph 1]; Methods [Study setting and Design, paragraphs 1-5] | | | | |
| Setting | 5 | | | Describe the setting, locations, and relevant dates, including periods of recruitment, exposure, follow-up, and data collection | OK: Methods [Study setting and Design, paragraphs 1-2] | | | | |
| Participants | 6 | | | (*a*) *Cohort study*—Give the eligibility criteria, and the sources and methods of selection of participants. Describe methods of follow-up  *Case-control study*—Give the eligibility criteria, and the sources and methods of case ascertainment and control selection. Give the rationale for the choice of cases and controls  *Cross-sectional study*—Give the eligibility criteria, and the sources and methods of selection of participants | OK: Methods [Study setting and Design, paragraph 2] | | | | |
|  |  |  |  | (*b*)*Cohort study*—For matched studies, give matching criteria and number of exposed and unexposed  *Case-control study*—For matched studies, give matching criteria and the number of controls per case | Not applicable | | | | |
| Variables | 7 | | | Clearly define all outcomes, exposures, predictors, potential confounders, and effect modifiers. Give diagnostic criteria, if applicable | OK: Methods [Autopsy procedures, paragraph 1]; Methods [Histological and microbiological analyses, paragraphs 1-2]; Methods [Determination of cause of death, paragraphs 1-3]; Methods [Definitions and statistical methods, paragraphs 1-2] | | | | |
| Data sources/ measurement | 8* | | | For each variable of interest, give sources of data and details of methods of assessment (measurement). Describe comparability of assessment methods if there is more than one group | The only variables used in the study are the CoD established by MIA and CDA. The sources and details of the data are given in the manuscript [See Methods section] | | | | |
| Bias | 9 | | | Describe any efforts to address potential sources of bias | Both the MIA and the CDA include a degree of subjective expert interpretation. In this study both evaluations were made by the same group of experts in order to minimize differences in expertise between observers (See Methods [Histological and microbiological analyses, paragraphs 1-2]; [Determination of cause of death, paragraph 1] | | | | |
| Study size | 10 | | | Explain how the study size was arrived at | Sample size was determined before starting the study, and it is included in the study protocol. The sample size calculation was performed for the primary proposal of the study, based on the comparison of the sensitivity of the MIA to detect the same diagnosis as the CDA. To calculate an adequate and feasible sample size for the validation of the MIA technique in comparison to CDA, we applied the approach proposed by Li and Fine (Li J, Fine J. On sample size for sensitivity and specificity in prospective diagnostic accuracy studies. Statistics in medicine 2004; 23:2537-50) to compare the sensitivity of two tests on the same subject (i.e. in our case MIA and CDA). | | | | |
| Quantitative variables | 11 | | | Explain how quantitative variables were handled in the analyses. If applicable, describe which groupings were chosen and why | Not applicable. No quantitative variables | | | | |
| Statistical methods | 12 | | | (*a*) Describe all statistical methods, including those used to control for confounding | OK: Methods [Definitions and statistical methods, paragraphs 1-2] | | | | |
|  |  |  |  | (*b*) Describe any methods used to examine subgroups and interactions | Not applicable | | | | |
|  |  |  |  | (*c*) Explain how missing data were addressed | Not applicable | | | | |
|  |  |  |  | (*d*) *Cohort study*—If applicable, explain how loss to follow-up was addressed  *Case-control study*—If applicable, explain how matching of cases and controls was addressed  *Cross-sectional study*—If applicable, describe analytical methods taking account of sampling strategy | Not applicable | | | | |
|  |  |  |  | (*e*) Describe any sensitivity analyses | Not applicable | | | | |
| Results | | | | |  | | | | |
| Participants | | 13* | (a) Report numbers of individuals at each stage of study—eg numbers potentially eligible, examined for eligibility, confirmed eligible, included in the study, completing follow-up, and analysed | | | | OK: Results [Characteristics of stillbirths and neonatal deaths, paragraph 1] |  |  |
|  |  |  | (b) Give reasons for non-participation at each stage | | | | OK: Methods [Study setting and Design, paragraph 2] |  |  |
|  |  |  | (c) Consider use of a flow diagram | | | | Not applicable |  |  |
| Descriptive data | | 14* | (a) Give characteristics of study participants (eg demographic, clinical, social) and information on exposures and potential confounders | | | | OK: Results [Characteristics of stillbirths and neonatal deaths, paragraph 1] |  |  |
|  |  |  | (b) Indicate number of participants with missing data for each variable of interest | | | | Not applicable |  |  |
|  |  |  | (c) *Cohort study*—Summarise follow-up time (eg, average and total amount) | | | | Not applicable |  |  |
| Outcome data | | 15* | *Cohort study*—Report numbers of outcome events or summary measures over time | | | | Not applicable |  |  |
|  |  |  | *Case-control study—*Report numbers in each exposure category, or summary measures of exposure | | | | Not applicable |  |  |
|  |  |  | *Cross-sectional study—*Report numbers of outcome events or summary measures | | | | Not applicable |  |  |
| Main results | | 16 | (*a*) Give unadjusted estimates and, if applicable, confounder-adjusted estimates and their precision (eg, 95% confidence interval). Make clear which confounders were adjusted for and why they were included | | | | OK: Methods [Determination of cause of death, paragraphs 1-3]; [Definitions and statistical methods, paragraphs 1-2] |  |  |
|  |  |  | (*b*) Report category boundaries when continuous variables were categorized | | | | Not applicable |  |  |
|  |  |  | (*c*) If relevant, consider translating estimates of relative risk into absolute risk for a meaningful time period | | | | Not applicable |  |  |
| Other analyses | | 17 | Report other analyses done—eg analyses of subgroups and interactions, and sensitivity analyses | | | | No other analyses done |  |  |
| Discussion | | | | |  | | | | |
| Key results | | 18 | Summarise key results with reference to study objectives | | | | OK: Discussion [paragraphs 1-5] |  |  |
| Limitations | | 19 | Discuss limitations of the study, taking into account sources of potential bias or imprecision. Discuss both direction and magnitude of any potential bias | | | | OK: Discussion [paragraph 6] | |  |
| Interpretation | | 20 | Give a cautious overall interpretation of results considering objectives, limitations, multiplicity of analyses, results from similar studies, and other relevant evidence | | | | OK: Discussion [paragraph 1] | |  |
| Generalisability | | 21 | Discuss the generalisability (external validity) of the study results | | | | OK: Conclusion [paragraph 1] | |  |
| Other information | | | | |  | | |  |  |
| Funding | | 22 | Give the source of funding and the role of the funders for the present study and, if applicable, for the original study on which the present article is based | | | OK: Submission form | |  |  |
